# Prevalence of internet addiction and anxiety, and factors associated with the high level of anxiety among adolescents in Hanoi, Vietnam during the COVID-19 pandemic

**DOI:** 10.1186/s12889-023-17348-2

**Published:** 2023-12-06

**Authors:** Tran Minh Dien, Pham Thi Lan Chi, Pham Quang Duy, Le Ha Anh, Nguyen Thi Kim Ngan, Vu Thi Hoang Lan

**Affiliations:** 1 Vietnam National Children’s Hospital, Dong Da, Hanoi, Vietnam; 2 Faculty of Sciences, University of British Columbia, Vancouver, BC Canada; 3 Hanoi Amsterdam High School for the gifted, Hanoi, Vietnam; 4 The University of North Carolina, Vietnam Office, Hanoi, Vietnam; 5 Faculty of Fundamental Sciences, Hanoi University of Public Health, No. 1A Duc Thang Ward, North Tu Liem, Hanoi, Vietnam

**Keywords:** Anxiety, Adolescents, COVID-19, Internet addiction, Vietnam

## Abstract

**Background:**

The COVID-19 pandemic and the resulting isolation measures created an increase in the usage of smart devices and internet among adolescents. This study aims to estimate the prevalence of internet addiction, the prevalence of high level of anxiety as well as to examine factors associated with the high level of anxiety among adolescents in Hanoi, Vietnam during the COVID-19 pandemic.

**Method:**

Data was collected using respondent-driven sampling and Google online survey forms from a sample of 5,325 school students aged 11–17 in Hanoi between October and December 2021. A short scale consisting of 5 items was used to measure internet addiction and the GAD-7 was used to measure adolescent anxiety level.

**Results:**

The findings revealed that 22.8% and 7.32% of adolescents experienced moderate and severe anxiety. About 32.7% of the study sample exhibited at least three internet addiction indicators. Logistic regression analysis identified significant predictors for high levels of adolescent anxiety. Being female, family experiencing economic difficulties, and exposure to domestic violence were associated with higher risk of anxiety disorder (OR 1.78, 1.45, and 2.89, respectively). Both average daily online time and internet addiction demonstrated gradient association with high level of anxiety.

**Conclusion:**

The prevalence of internet addiction and high level of anxiety were high among adolescents in Hanoi, Vietnam during the COVID-19 pandemic. The study highlights the importance of implementing measures at the family and school levels to promote a balanced and healthy approach to smart device use among adolescents.

## Introduction

Adolescence is generally defined as the transitional period between childhood and adulthood. This period is characterized by significant physical, cognitive, emotional, and social changes as individuals navigate their way towards adulthood [1]. COVID 19 pandemic is a very stressful period for adolescents due to uncontrollable stressors such as school closure, lack of social connections, fear of diseases or changes in social and family settings [2]. As adolescents in transitional period, the psychological impacts of pandemic on them may be more significant than on adults because they are more vulnerable [3]. Anxiety disorder is one of the most prevalent mental health challenges adolescents faces during pandemic time. One study from China reported that 22.6% of children reported any depressive symptoms and 18.9% of children reported anxiety symptoms [4]. Another study also in China among adolescents indicated that the prevalence of depressive and anxiety symptoms was 43.7% and 37.4%, respectively [5].

Another factor that may profoundly affect adolescents’ anxiety during COVID-19 era was excessive internet use. In the modern digital era, the internet has become an integral part of adolescent’s lives, affecting their social interactions, academic pursuits, and personal growth and can even cause internet addiction.

Internet addiction refers to an uncontrollable urge to spend excessive time online, often leading to neglect of responsibilities, withdrawal symptoms when attempting to reduce internet use, and a loss of interest in other activities [6]. For the general population, social isolation strategies for controlling the pandemic had led to increased Internet use and increased risk for internet addiction. One study in China reported that the overall prevalence of Internet addiction among general population was 36.7%. [7].

Previous studies had shown that adolescents are particularly vulnerable to excessive internet use, leading to negative consequences in various aspects of their life, especially mental health. Adolescents who struggle with internet addiction are more likely to experience various mental health issues such as anxiety, depression, loneliness, and low self-esteem [8, 9]. Excessive internet use can lead to a lack of real-life social interactions, contributing to feelings of isolation and withdrawal. During Covid-19, the situation of internet addiction may be even worse for adolescents due to the additional effect of online learning activities. One study in Vietnam reported that more than 25% adolescents spent minimum 8 h/day online [9]. Multiple previous studies reported that globally, adolescents of varying backgrounds experience higher rates of anxiety, depression, and stress due to the pandemic [2, 10]. Current literature presents a paucity of recent data pertaining to the prevalence of internet addiction among adolescents during the COVID-19 pandemic era. Moreover, limited research has addressed the compounded impact of internet addiction and the resultant social isolation arising from the pandemic, particularly in the context of exacerbating adolescent anxiety. It is important to quantify the impact of these cumulative effects to provide support systems and resources to help adolescents cope with the stressors associated with COVID-19. This manuscript aims to estimate the prevalence of internet addiction, the prevalence of high level of anxiety as well as to examine factors associated with the high level of anxiety among adolescents in Hanoi, Vietnam during the COVID-19 pandemic.

## Methodology

### Study design and sample

This study employed a cross-sectional design. Study subjects included students aged 11–17 years old at some secondary and high schools in Ha Noi, Viet Nam. Using Google online survey forms to collect data, the survey was facilitated through various channels such as social media, emails, school websites, teachers, parents and school clubs. The study employed a sample of 5,325 students (i.e., 2100 students from secondary schools and 3225 from high schools). Both parents and students were provided with a consent form that explained the study objectives and the data collection process in detail. Students were only allowed to complete the Google form after obtaining permission from their parents.

The inclusion criteria were students aged 11–17 years residing in Hanoi’s rural or urban areas during the period between October and December 2021. Exclusion criteria pertained to students who did not obtain parental permission to complete the Google form.

### Data collection

Data was collected through a monkey survey in Google Forms. The electronic questionnaire’s content and interface were designed and tested so that the audience could answer on media such as smartphones, tablets, and computers. Participants completed the online survey between October and December 2021. The survey included demographic factors, hardships during the COVID-19 pandemic, family care, family pressure, and coping strategies.

### Measurements

#### Internet use

Measuring the daily average online time of an adolescent, categorized into four groups: less than 4 h, 4–6 h, 6–8 h, and 8 h and more.

#### Anxiety

The Generalized Anxiety Disorder Assessment (GAD-7) was used. It was a seven-item instrument that is used to measure or assess the severity of generalized anxiety disorder (GAD). Each item asked the individual to rate the severity of his or her symptoms over the past two weeks. Response options included “Not at all”, “Several days”, “More than half the days” and “nearly every day”. GAD-7 total score for the seven items ranges from 0 to 21. Scores of 5, 10, and 15 are designated as the threshold values for categorizing anxiety levels as mild, moderate, and severe, respectively [11]. For the descriptive analysis, the study presented results for all cut off points. A study in Asian reported that a cut-off score of 7 should indicate a potential diagnosable condition in Asian adolescents, thus we also examined this cut off point [12]. For the logistics regression model, as previous studies recommended the cutoff point of 10 for further intervention when using GAD-7 as a screening instrument, the study outcome was defined as high level of anxiety (GAD7 total score is greater or equal to 10 or moderate/severe anxiety level) [13].

#### Internet addiction

The items measuring internet addiction were adapted from the Internet Addiction Diagnostic Questionnaire (IADQ) [14]. Because the original survey consists of multiple components, the study employed 5 short items measuring internet addiction: (1) Can not live without internet and smart devices? (2) Often lie to parents about online time; (3) Use internet to cope with stress/undesired things? (4) Being angry when parents control online time and 5) Prefer to spend time on internet than family/friends. Response option included “Yes” and “No”. The total score for 5 items ranges from 0 to 5.

Three stressors introduced by Covid 19 were examined: Ever experienced your family having difficulty in buying food, Experience family domestic violence during COVID time and Have at least one of your parents’ unemployment due to COVID.

***Other information*** collected was demographic information (gender, age), grade, and the person with whom children live (with parents, with a single parent, and with no one).

### Data analysis

In this study, the prevalence of internet addiction and stress among the whole sample size and for subgroups were estimated using frequency and crosstab techniques. Multiple logistics regression was applied to examine factors associated with adolescent’s high level of anxiety. The study outcome for the logistics regression model was high anxiety level (i.e., GAD7 score > = 10). In the multivariate model, due to the small proportion of adolescents with 5 indicators of internet addiction, we had combined two categories, 4 and 5 indicators into 1 group (i.e., 4 or more indicators). A p-value < 0.05 was regarded as statistically significant. Statistical analyses were performed using STATA version 17.0.

### Ethics

Ethics approval, including the confidentiality of participants’ consents and information, was approved by the Human Research Ethics Committee at Hanoi University of Public Health (Approval No. #382/2021/YTCC/HD3). All methods were carried out in accordance with relevant guidelines and regulations. Voluntary participation and informed consent were obtained electronically from all respondents, and their parents/ guardians when they were under 18 years before completing the online survey. The respondents and their parents/guardians were provided with comprehensive details about the study, enabling them to make an informed decision about their involvement. Respondents were also given contact information should they have any questions about the study after they completed the survey. All data were kept in encrypted software, and no personal identifiers were used.

## Results

### Characteristics of the study sample

Table 1 presents the sociodemographic characteristics of the participants. The study included a total of 5,315 children aged 11 to 17 years, consisting of 2,177 boys (41.0%) and 3,138 girls (59.0%). Most participants (92%) reported living with their parents, while 7.6% lived with a single parent, and 0.4% reported living alone. Approximately 53% of the participants resided in rural areas of Ha Noi.

Regarding the stressors introduced by COVID-19 pandemic, around 27.3% of participants living in families reported experiencing difficulties in purchasing food during this period. Additionally, a quarter of the participants had at least one parent who was unemployed due to the pandemic. Approximately 12% of respondents reported ever experiencing domestic family violence during the COVID-19 period. More than a quarter of the participants spent more than 8 h online per day.

Table 1 also displays the status of five indicators used to measure internet addiction. The findings revealed that 54.7% of adolescents acknowledged their inability to live without smart devices/internet. Moreover, a significant proportion of 58.1% reported resorting to smart devices and the internet as a coping mechanism for stress or unwanted events. The proportion of study subjects with no indicator of addiction (i.e., answer No to all questions) was only 13.8% while the proportion of adolescents exhibited at least 3 indicators was 32.7%.

**Table 1:** Characteristics of the study population

Factor	n (%)
Age (mean/SD)	14.8 (1.7)
11–13 years old	1176 (22.4%)
14–17 years old	4068 (77.6%)
Gender	
Male	2132 (40.7%)
Female	3112 (59.3%)
Location	
Rural	2765 (52.7%)
Urban	2479 (47.3%)
Live with	
With both parents	4824 (92.0%)
With single parent	401 (7.6%)
With no one	19 (0.4%)
Average online time per day	
less than 4 h	2204 (42.0%)
4 to 6 h	439 (8.4%)
6 to 8 h	1169 (22.3%)
8 h or more	1432 (27.3%)
Ever experienced your family having difficulty in buying food	1431 (27.3%)
Experience family domestic violence during COVID time	604 (11.5%)
Have at least one of your parents’ unemployment due to COVID	1269 (24.2%)
Internet addiction	
Often lie parents about time to use internet	681 (13.0%)
Often use smart devices to cope with stress/undesired things	3046 (58.1%)
Being angry when parent control smart devices	1306 (24.9%)
Prefer to spend time on smart devices than family/friends	2242 (42.8%)
Can not live with smart devices/internet	2,869 (54.7%)

### Levels of anxiety measured by GAD-7

Table 2 presents the prevalence of four levels of anxiety measured by GAD-7. The % of adolescents with severe anxiety (GAD-7 total score greater than 14) was 7.32%. About 22.8% participants reported a moderate level of anxiety (GAD-7 score from 10 to 14). In total, 30% of the study sample demonstrated the needs for intervention because their score in the moderate to severe range. Using the cut off point of 7 for Asian, the prevalence of adolescents with potential diagnosable condition would be 59.4%. The average GAD7 score of the study sample was 7.62(Sd = 4.5).

**Table 2:** Anxiety levels measured by GAD-7

Anxiety level	n (%)
Minimum anxiety (Total score 0–4)	1,285 (24.5%)
Mild (Total score 5–9)	2,381 (45.4%)
Moderate (Total score 10–14)	1,194 (22.8%)
Severe (Total score > 14)	384 (7.3%)
Total GAD 7 score (mean/SD)	7.62 (4.5)

### Prevalence of high level of anxiety by subgroups of the study sample

#### Prevalence of high level of anxiety and crude association of independent variables and high level of anxiety

Table 3 demonstrates the prevalence of high level of anxiety (i.e., GAD7 > = 10, indicating moderate to severe anxiety) by different independent variables, together with the crude OR and 95% CI. Significant ORs were reported for all variables, except location (ORs for Urban vs. Rural was 1.10, p > .05.

Two gradient associations with the outcomes were observed. Firstly, as daily online time increased, the prevalence of high-level anxiety also rose significantly, as indicated by statistically significant crude odds ratios (ORs). Secondly, as the total number of instances of internet addiction increased, the prevalence of high-level anxiety likewise exhibited an upward trend.

**Table 3:** Prevalence of adolescents with high level of anxiety among sub-groups of the study sample

	GAD7 < 10 (none/mild)		GAD7 > = 10 (moderate/severe)		Crude OR	95% CI
	n	%	n	%		
Age						
11–13 years old	1,656	77.67	476	22.33	1.00	
14–17 years old	2,010	64.59	1,102	35.41	1.49**	[1.28, 1.73]
Sex						
Male	895	76.11	281	23.89	1.00	
Female	2,771	68.12	1,297	31.88	1.91**	[1.68, 2.16]
Location						
Rural	1,959	70.85	806	29.15	1.00	
Urban	1,707	68.86	772	31.14	1.10	[0.98, 1.24]
Live with						
With parents	3,397	70.42	1,427	29.58	1.00	
With single parent	260	64.84	141	35.16	1.29*	[1.04, 1.60]
With no one	9	47.37	10	52.63	2.65*	[1.07, 6.52]
Online time						
less than 4 h	1,770	80.31	434	19.69	1.00	
4 to 6 h	330	75.17	109	24.83	1.35*	[1.06, 1.71]
6 to 8 h	760	65.01	409	34.99	2.19**	[1.87, 2.57]
8 h or more	806	56.28	626	43.72	3.17**	[2.73, 3.67]
Internet addiction						
None	581	80.25	143	19.75	1.00	
One indicator	1,048	77.12	311	22.88	1.21	[0.97, 1.51]
Two indicators	1,012	70.03	433	29.97	1.74**	[1.40, 2.15]
Three indicators	663	62.84	392	37.16	2.40**	[1.92, 3.00]
Four or more indicators	362	54.77	299	45.23	3.36**	[2.64, 4.26]
Ever experienced your family having difficulty in buying food						
No	2,772	72.70	1,041	27.30	1.00	
Yes	894	62.47	537	37.53	1.60**	[1.41, 1.82]
Experience family domestic violence during COVID time						
No	3,412	73.53	1,228	26.47	1.00	
Yes	254	42.05	350	57.95	3.83**	[3.22, 4.56]
Have at least one of your parents unemployment due to COVID						
No	2,828	71.14	1,147	28.86	1.00	
Yes	838	66.04	431	33.96	1.27**	[1.11, 1.45]

*p < .05. **p < .01. ***p < .001* Adjusted association between internet addiction and adolescent’s anxiety*

Table 4 presents the results of a multivariate logistic regression model analyzing the likelihood of experiencing high anxiety (i.e., moderate to severe anxiety measured by GAD7) among the study subjects. Five variables demonstrated a significant association with the outcome at a significant level of 0.05. Specifically, the odds ratio for females compared to males was 1.78 (95% CI: 1.56–2.03), indicating that female adolescents have a higher likelihood of experiencing high anxiety levels compared to males. Adolescents who reported their families’ experiencing difficulties in buying food had an odds ratio of 1.45 (95% CI: 1.26–1.67), suggesting that they faced a higher risk of experiencing elevated anxiety levels compared to other adolescents.

Furthermore, adolescents who reported experiencing domestic violence within their families during the COVID-19 period were at a higher risk of having high anxiety levels (OR = 2.89, p < .05) when compared to those whose families had no history of domestic violence.

After controlling all socio-demographic factors and three stressors associated with the COVID-19 period, both average daily online time and internet addiction exhibited a dose-response relationship with high anxiety levels. Specifically, compared to adolescents who spent less than 4 h online, those who spent 4–6 h, 6–8 h, and 8 h or more had odds ratios of 1.33, 1.76, and 2.28, respectively (all p < .05). As the total number of internet addiction indicators increased, the odds ratio for experiencing high anxiety levels also increased. However, statistically significant odds ratios were observed only for the “three indicators” and “four or more indicators” groups.

Three variables (i.e., age, location, and live with) showed significant associations in the crude association. However, when taking into other variables in the multivariate model, the adjusted ORs for these two variables became non-significant (p > .05).

**Table 4:** Multivariate model for having moderate/severe anxiety among adolescents

	Coefficient	p-value	95% CI
Average online time per day (ref: less than 4 h)			
4 to 6 h	1.33*	0.03	[1.04, 1.70]
6 to 8 h	1.76**	0.00	[1.46, 2.12]
8 h or more	2.28**	0.00	[1.89, 2.74]
Internet addiction (ref: no indicator)			
One indicator	1.00	0.97	[0.79, 1.26]
Two indicators	1.17	0.19	[0.92, 1.48]
Three indicators	1.31*	0.04	[1.01, 1.69]
Four or more indicators	1.44*	0.01	[1.09, 1.91]
Age (14–17 years old vs. 11–13 years old)	1.09	0.29	[0.93, 1.28]
Sex (Female vs. Male)	1.78**	0.00	[1.56, 2.03]
Urban vs. Rural	0.99	0.89	[0.87, 1.12]
Living with (Ref: with both parents)			
Single parent	1.17	0.16	[0.94, 1.47]
No one	2.26	0.10	[0.87, 5.88]
Ever experienced your family having difficulty in buying food (Yes vs. No)	1.45**	0.00	[1.26, 1.67]
Experience family domestic violence during COVID time (Yes vs. No)	2.89**	0.00	[2.40, 3.47]
Have at least one of your parents unemployment due to COVID (Yes vs. No)	1.10	0.21	[0.95, 1.27]
Intercept	0.12**	0.00	[0.09, 0.16]

*p < .05. **p < .01. ***p < .001

## Discussion

Anxiety is a global health concern, impacting one-fifth of all young people at some point in their lives [15]. This study employed The GAD-7 scale, one of the most widely used and well-validated self-report scales for anxiety disorder [11]. The study showed that overall, 30% of adolescents in the sample experience high level of anxiety (GAD-7 total score > = 10) and 7.33% of the sample exhibited severe anxiety. If using the cut off points of 7 for Asia suggested by Hang et al., in Hongkong sample [12], the prevalence of adolescents with potential diagnosable condition in this study can go up to 59.4%. While direct comparison with other studies was challenging because multiple issues such as different research tools, diagnostic criteria, study population can create a wide range of reported prevalence rates [15], results highlight the importance of addressing anxiety-related issues among adolescents and underscore the necessity of early identification and intervention strategies for adolescents experiencing anxiety in Vietnam.

Results indicated that only 13.8% of the study sample reported having no indicators of addiction, indicating that most adolescents exhibited some form of addictive behavior. More significant, approximately one in every three adolescents (32.7%) displayed at least three indicators of addiction. Over a quarter of study subjects spend more than 8 h online per day. While the usage of smart devices and the internet is inevitable and offers certain benefits, the results presented in this study collectively highlight a significant reliance on smart devices among the participants. Previous studies suggested potential negative consequences of internet addiction on various aspects, such as interpersonal relationships, emotional well-being, and stress management [16, 17]. It is crucial for future studies and interventions to delve deeper into these patterns to gain a better understanding and develop strategies aimed at promoting healthy and balanced smart device usage among adolescents.

Understanding the connection between internet addiction and anxiety disorder in adolescents is important. This study also examined the impact of internet addiction on adolescents’ anxiety by using a multivariate logistics regression model. The model incorporated four socio-demographic factors (age, gender, location, and living arrangement), three COVID-19 stressors (food scarcity, parental unemployment, and family violence during the COVID-19 period), and two variables related to internet usage (average daily internet time and total number of internet addiction indicators). Firstly, the study found a significant gender difference in anxiety levels, with females having a higher likelihood of experiencing high anxiety compared to males. The odds ratio of 1.78 suggests that female adolescents are at a greater risk of developing elevated anxiety levels. Previous studies had attributed the greater risk of developing high anxiety level in female adolescent to a combination of many factors. Biological changes, including hormonal fluctuations like estrogen and progesterone, can impact mood regulation or the developing adolescent brain, especially the prefrontal cortex, may render females more susceptible to anxiety. Sociocultural pressures play a substantial role, with social and peer pressures, gender role expectations, and societal standards affecting females more significantly. Coping mechanisms also differ, as females may ruminate or internalize stress, increasing their vulnerability to anxiety [18, 19].

COVID 19 pandemic resulted increasing domestic violence and economic difficulties [20]. These stressors would also create impacts on adolescent anxiety. Results revealed a strong association between experiencing domestic violence within the family during the COVID-19 period and high anxiety levels. Adolescents who reported such experiences had an odds ratio of 2.89, indicating a significantly increased risk of elevated anxiety. Previous studies showed that the psychological impact of family domestic violence on adolescents was multifaceted. It could disrupt their emotional development, instill maladaptive coping strategies, and create an environment of chronic stress and fear, all of which can significantly increase their vulnerability to anxiety disorders [21–24]. Another significant factor associated with high anxiety levels is the experience of family difficulties in buying food. Adolescents who reported such difficulties had an odds ratio of 1.45, indicating a higher risk of experiencing elevated anxiety levels. The experience of family difficulties in buying food can lead to anxiety among adolescents due to the pervasive sense of insecurity, stress within the family, physical and cognitive impacts of malnutrition, social pressures, academic concerns, and worries about the future [25–27]. These findings highlight the vulnerable groups with urgent need for prioritizing interventions, adolescents with low socio-economic family and adolescents with domestic violence. Addressing food insecurity and providing support to families facing financial difficulties can be crucial in alleviating anxiety levels among affected adolescents [28].

Controlling for socio-demographic factors and COVID-19 stressors, both online time and internet addiction showed gradient relationships with adolescent’s high anxiety level. The OR for having high anxiety raised up as the average online time and the number of internet addiction increased, indicating strong evidence about the adverse impacts of excessive internet use on elevated anxiety levels among adolescents. In term of quantifying the impact of online time, the adjusted ORs for online time > 8 h was 2.28 (p < .05), indicating that the odds of having high levels of anxiety was more than twice higher among adolescents use internet more than 8 h per day compared to adolescent with less than 4 h per day. When examining the crude association, the prevalence of high anxiety among adolescents with 4 or 5 indicators of internet addiction was more than two times higher than that among adolescents with no indicator. However, when adjusting for other variables in the multivariate model, the adjusted association remained statistically significant, but the magnitude got smaller (ORs = 1.44). Previous studies had suggested that spending excessive time on the internet can lead to social isolation, increase feelings of loneliness, which is associated with higher levels of anxiety [16, 17, 29]. Excessive internet use exposes adolescents to cyberbullying and online harassment, which can have severe psychological consequences [30]. Constant exposure to such online stressors can increase anxiety levels, especially when adolescents lack the necessary coping mechanisms to manage the influx of distressing information [16, 17]. Other demographic factors such as age, location or whom children live with did not have significant impacts on the study outcome after adjusting for other covariates such as COVID19 stressor, online time and internet addiction.

It is important to acknowledge the limitations of the study, such as potential confounding factors or unmeasured variables such as pre-existing vulnerabilities, adolescent coping skills that could influence the observed associations. Additionally, the cross-sectional nature of the study limits the ability to establish causality. Further longitudinal research is needed to examine the temporal relationships between internet addiction, anxiety, and associated factors. These findings provide valuable insights for developing targeted interventions aimed at reducing anxiety levels, particularly among females, individuals facing socio-economic challenges, and those exposed to domestic violence. Families and schools need to implement measures to promote a balanced and healthy approach to smart device usage among adolescents. Mental health support can be beneficial for adolescents who are struggling with device addiction and its impact on their mental well-being.

## Conclusion

The prevalence of adolescents in Hanoi, Vietnam with high level of anxiety during COVID-19 pandemic was significant. Multiple factors associated with adolescents’ high level of anxiety reported in this study were gender, family domestic violence, food insecurity, excessive online time and internet addiction. Through understanding the intricate connection between internet addiction and anxiety disorder, this study provides information to develop interventions, policies, and educational programs that encourage responsible internet use and protect the well-being of young individuals in the digital age.

## Data Availability

The datasets used and/or analyzed during the current study are available from the corresponding author on reasonable request.
